# Development and validation of sustainable employability index among older employees

**DOI:** 10.1093/occmed/kqac120

**Published:** 2023-02-14

**Authors:** S Neupane, P K C, S Kyrönlahti, A Siukola, H Kosonen, K Lumme-Sandt, P Nikander, C H Nygård

**Affiliations:** Unit of Health Sciences, Faculty of Social Sciences, Tampere University, Tampere FI-33014, Finland; Gerontology Research Center, Tampere University, Tampere FI-33014, Finland; Unit of Health Sciences, Faculty of Social Sciences, Tampere University, Tampere FI-33014, Finland; Gerontology Research Center, Tampere University, Tampere FI-33014, Finland; Department of Public Health, University of Turku and Turku University Hospital, Turku FI-20014, Finland; Stress Research Institute, Department of Psychology, Stockholm University, Stockholm SE-10691, Sweden; Unit of Health Sciences, Faculty of Social Sciences, Tampere University, Tampere FI-33014, Finland; Gerontology Research Center, Tampere University, Tampere FI-33014, Finland; Gerontology Research Center, Tampere University, Tampere FI-33014, Finland; Clinical Medicine, Faculty of Medicine and Health Technology, Tampere University, Tampere FI-33014, Finland; Unit of Health Sciences, Faculty of Social Sciences, Tampere University, Tampere FI-33014, Finland; Gerontology Research Center, Tampere University, Tampere FI-33014, Finland; Unit of Health Sciences, Faculty of Social Sciences, Tampere University, Tampere FI-33014, Finland; Gerontology Research Center, Tampere University, Tampere FI-33014, Finland; Gerontology Research Center, Tampere University, Tampere FI-33014, Finland; Unit of Health Sciences, Faculty of Social Sciences, Tampere University, Tampere FI-33014, Finland; Gerontology Research Center, Tampere University, Tampere FI-33014, Finland

## Abstract

**Background:**

Sustainable employability (SE) has become an important factor for keeping people in the labour market and enabling the extension of working life.

**Aims:**

We developed and validated an SE index to predict assured workability in 2 years. Additionally, we developed a scoring tool to use in practice.

**Methods:**

A questionnaire survey of postal employees aged ≥50 years was conducted in 2016 and followed up in 2018 (*n* = 1102). The data were divided into training and validation sets. The outcome was defined as whether the employees had an assured workability after 2 years or not. Multivariable log-binomial regression was used to calculate the SE index. The area under the curve (AUC) was calculated to assess the discriminative power of the index.

**Results:**

The probability of assured workability increased with increasing quintiles of the SE index. The highest quintiles of the SE index showed the highest observed and expected assured workability in 2 years. The predictive ability, area under the curve (AUC) for training was 0.79 (95% CI 0.75–0.83) and for validation data was 0.76 (95% CI 0.73–0.80). In the scoring tool, the self-rated health, workability, job satisfaction and perceived employment had the highest contribution to the index.

**Conclusions:**

The SE index was able to distinguish the employees based on whether they had assured workability after 2 years. The scoring method could be used to calculate the potentiality of future employability among late midlife postal employees.

## Teaser

We developed and internally validated a sustainable employability index to predict assured workability based on nine indicators that cover the health, well-being and employability characteristics of older employees. The tool could be used by occupational healthcare, in epidemiological research, and government programmes as a screening tool for employability among older employees and could ultimately help them to conduct feasible programmes targeting employees with suboptimal employability to improve their employability.

**Key learning points**

**What is already known about this subject:**

- Sustainable employability has become an important factor for keeping people in the labour market and enabling the extension of working life.
- Several definitions of sustainable employability exist in the literature but most of them lack a clear conceptual framework.
- Sustainable employability is a multidimensional concept which involves the health, well-being and employability characteristics of an individual and the sustainability of those characteristics by age.

**What this study adds:**

- An internally validated sustainable employability index to predict assured workability based on nine indicators among older postal employees.
- The sustainable employability index correctly predicted the assured workability of the respondents in 2 years, area under the curve for training data: 0.79 (95% confidence interval 0.75–0.83) and area under the curve for validation data: 0.76 (95% confidence interval 0.73–0.80).
- A scoring tool to estimate assured workability in which the self-rated health, workability, job satisfaction and perceived employment had the highest contribution.

**What impact this may have on practice or policy:**

- The sustainable employability index can be used as a practical tool to screen the potential employability of late midlife employees.

## Introduction

Policies for extended working lives have increased the need for sustainable employability (SE) [1]. SE is a multidimensional concept of the extent to which workers are able and willing to work now and in the future [2]. Although several definitions of SE exist in the literature [3–9], most of these definitions lack a clear conceptual framework based on potential indicators of SE. Fleuren *et al.* [3] presented a more comprehensive definition which covers functioning in the workforce as a multidimensional construct which includes three domains; health, well-being and employability. They also [10] presented indicators of SE as an outcome of or affected by aspects of employment characteristics; perceived health status, workability, fatigue, need for recovery, job satisfaction, motivation to work, employability, skill gap and job performance.

Potential indicators of SE and their changes were recently studied during a 2-year follow-up among postal service workers aged 50 years and above. The indicators showed consistency with no significant change over time [11]. In total, nine indicators adapted from previous definitions of SE, representing three main domains were studied: self-rated health, workability, time and resources and recovery after work, representing health; job satisfaction and motivation to work, representing well-being; and perceived employment, enough on-the-job training and relevance of job, representing employability. To our knowledge, no earlier study has developed a validated practical SE index. The SE index presented here is based on limited but important indicators and offers evidence-based input to inform policy practice for predicting assured workability in 2 years, among a cohort of postal service employees aged 50 years and above. Additionally, a scoring method for use in practice was developed.

## Methods

The analyses were performed using data from a prospective follow-up survey. The baseline questionnaire survey was conducted among employees of the Finnish postal services in 2016 and was followed in 2018 [11]. A questionnaire was sent to all Finnish postal service workers aged ≥50 years in 2016 and 44% (*n* = 2096) replied to the survey. The follow-up survey was completed with a 76% response rate from baseline respondents (*n* = 1466). This study utilized data from 1102 subjects who replied to both the baseline and the follow-up surveys and had complete information on each of the measures used in the analysis.

The study was approved by the Academic Ethics Committee of Tampere Region (ethical approval number: 32/2016).

The outcome variable ‘assured workability in 2 years’ was measured in the follow-up survey. Employees were asked to estimate their workability in present work from the point of view of health after 2 years on a scale of 0 (I can’t) to 10 (pretty sure). Based on the literature [12,13], as well as the distribution of the variable (top-third) in our data, a dichotomous variable was created as having assured ability (8–10) versus no assured workability (0–7).

The nine indicators of SE were measured at baseline on an ordinal scale and were used as continuous variables in the analysis.

Employees were asked to rate their health from the point of view of work on a response scale of 0 (extremely bad) to 10 (extremely good) [14].

Workability was based on the first item of the workability index (WAI), which was assessed with a single-item question ‘workability at present compared to lifetime best’ on a scale of 0–10 [15,16]. The single-item question is validated against the full WAI and can be used as a reasonable alternative [12,17].

The question ‘Do you have enough time and resources for your friends and hobbies?’ with the response option of 0 (hardly) to 10 (totally) was used to measure the time and resources for friends and hobbies [14].

Employees were asked ‘how well do you feel you recover from the workload after a day/shift of work?’ with the response options 0 (very poorly) to 10 (very well) [18].

Employees were asked ‘how satisfied are you with your current job?’ with response options forming a scale of 1–6 where 1 = very satisfied, 2 = nearly satisfied, 3 = neither satisfied nor dissatisfied, 4 = quite dissatisfied, 5 = very dissatisfied and 6 = can’t say [18]. To make the measures consistent with other indicators, we reversed the scale and removed those who replied, ‘can’t say’. The new score is on a scale of 1–5, where 1 = very dissatisfied and 5 = very satisfied.

The employees were asked about their motivation at work on a scale of 0 (not at all) to 10 (very much) [14].

Perceived employment was measured as ‘If you now became unemployed, do you think you would get a new job which corresponds to your profession and work experience?’ with the response options 1–5, where 1 = yes, sure, 2 = probably yes, 3 = probably not, 4 = surely not and 5 = cannot say [18]. For this analysis, we reversed the scale and also removed those who replied, ‘cannot say’. The new say is on a scale of 1–4, where 1 = surely not and 4 = sure, yes.

Employees were asked if they get enough training to support their job on a scale from 0 (not at all) to 10 (enough) [14].

Employee’s experience of doing important and significant work for the company (relevance of job) was measured on a scale of 1–6, where 1 = daily, 2 = weekly, 3 = monthly, 4 = less often, 5 = never and 6 = cannot say [19]. For this analysis, we reversed the scale and also removed those who replied, ‘cannot say’. The new say is on a scale of 1–5, where 1 = never and 5 = daily.

Other baseline characteristics used in the study were: age in years, gender (male, female), work experience in years, an education level (basic education, college-level training, academic degree and others), occupational class (white-collar or blue-collar), work shifts (regular daywork or two shifts including night work) and marital status (married/living together or single/others).

We first randomly divided the data into training and validation sets in a 1:1 ratio balancing by age and gender. We fitted a log-binomial regression model for the dichotomous outcome ‘assured workability in 2 years to the training data using the set of nine variables from baseline. All the variables were added to the model simultaneously. The selection of these variables was made based on previous studies [3,11], which have shown that they can be used as valid indicators of SE. Variance inflation factors were used to investigate multicollinearity amongst predictors, with a value of >3 being an indicator of multicollinearity. We used pseudo-*R*^2^ and chi-square values for the goodness of model fit. The regression coefficients from the multivariable log-binomial regression model were then used to calculate an index as a linear combination of the estimates as follows:


$$\begin{array}{lll} & SE\;index= & \alpha +\;\{\beta_{1}\times (Self-rated\;health)+\beta_{2}\times (Work\;ability)+\beta_{3}\\ & \;\times (Time\;and\;resources)+\beta_{4}\times (Recovery\;after\;work)+\beta_{5} & \\ & \;\times (Job\;satisfaction)+\beta_{6}\times (Motivation\;at\;work)+\beta_{7} & \\ & \;\times (Perceived\;employment)+\beta_{8}\times (Enough\;training)+\beta_{9} & \\ & \;\times (Relevance\;of\;job)\} & \end{array}$$

where *α* = constant term estimated from the regression model; *β*_1_…*β*_9_ = regression coefficient estimated from the regression model.

The regression coefficients were converted into odds ratios (ORs) and presented with their 95% confidence intervals (CIs).

The SE index was then divided into quintiles. We calculated the predicted probability of having assured workability in 2 years by regressing SE index quintiles with the assured ability to work. The mean of the predicted probability of having assured workability was calculated for each quintile and the results are presented graphically.

The same set of variables was used in the multivariable log-binomial model from the validation data set. The SE index was also calculated from the validation set to avoid overfitting and overestimation of the predictive ability of the index. We calculated and compared the expected and observed probabilities for both the training and validation data sets.

Receiver operating characteristic was calculated for the training and validation data sets to illustrate the sensitivity and specificity of the SE index. The area under the curve (AUC) was calculated to assess the discriminative power of the index.

Furthermore, we calculated the score for each of the indicators based on the coefficients from the log-binomial model calculated from the training data. The scores were calculated by dividing each estimate by the sum of the estimates of all indicators and then multiplying by 100. The scores are rounded-up.

## Results

The mean age of the study population was 55.97 years (standard deviation (SD): 3.11) and 60% were male. In total, 538 subjects were selected for the training data set and 564 for the validation data (Table 1). About 33% of the employees in the training data set had assured workability in 2 years, while 42% of the employees in the validation set had that ability. The mean age of the employees with those having assured workability was about the same in both the training and validation data sets. Comparatively more male than female employees had assured ability in the validation data, while almost no difference was found in the training data. Other variables were similarly distributed in training data and validation data sets.

**Table 1.** Distribution of baseline characteristics of the study population in training and validation data set stratified by assured workability in 2 years (outcome variable)

Characteristics	Total (*N* = 1102)	Training (*n* = 538)		Validation (*n* = 564)	
		Assured workability in 2 years		Assured workability in 2 years	
		Yes = 179	No = 359	Yes = 240	No = 324
Age (mean, SD)	1102	55.5 (3.0)	56.4 (3.0)	55.3 (3.1)	56.3 (3.0)
Gender					
Female	440	71 (34)	140 (66)	86 (38)	139 (62)
Male	662	107 (33)	217 (67)	153 (45)	185 (55)
Work experience (years) (mean, SD)		26.6 (11.0)	29.0 (9.7)	27.1 (11.4)	28.7 (10.8)
Education					
Basic school	504	68 (28)	173 (72)	109 (41)	154 (59)
College level	335	64 (37)	107 (63)	61 (38)	100 (62)
Academic degree	52	13 (59)	9 (41)	23 (77)	7 (23)
Others	206	33 (32)	69 (68)	46 (44)	58 (56)
Occupational class					
White-collar	152	36 (51)	35 (49)	45 (56)	36 (44)
Blue-collar	950	143 (31)	321 (69)	195 (40)	288 (60)
Work shifts					
Regular daywork	788	132 (35)	249 (65)	165 (41)	239 (59)
Two shifts or other types	314	46 (29)	109 (71)	75 (35)	85 (66)
Marital status					
Married/living together	805	137 (35)	260 (66)	172 (42)	234 (58)
Single/others	295	42 (31)	99 (69)	64 (41)	89 (59)

The indicators show a similar type of association with the outcome in both the training and validation data sets (Table 2). However, only three indicators (self-rated health, workability and enough on-the-job training) in the training data set and three indicators (workability recovery after work, and perceived employment) in the validation data set were statistically significantly associated with the assured workability in 2 years. The magnitude of the association was highest for self-rated health (OR 1.41, 95% CI 1.10–1.80) in the training data. Workability was the only variable that was statistically significantly associated with the assured workability in 2 years in both the training and validation data sets. Recovery after work was positively associated with having assured workability in 2 years (OR 1.17, 95% CI 1.04–1.32) in the validation data set, while in the training data the magnitude of having enough on-the-job training had lower odds of assured workability in 2 years (OR 0.91, 95% CI 0.85–0.99).

**Table 2.** Estimates (OR) from multivariable log-binomial regression model with their 95% CI for the indicators of employability index for assured workability after 2 years in the training and validation data sets

Employability indicators	OR (95% CI)	
	Training	Validation
Self-rated health	1.41 (1.10–1.80)	1.20 (0.98–1.46)
Work ability	1.30 (1.08–1.56)	1.24 (1.05–1.46)
Time and resources	1.06 (0.95–1.19)	1.03 (0.94–1.13)
Recovery after work	1.07 (0.94–1.22)	1.17 (1.04–1.32)
Job satisfaction	1.26 (0.93–1.70)	1.07 (0.82–1.41)
Motivation at work	1.02 (0.88–1.18)	0.94 (0.82–1.07)
Perceived employment	1.23 (0.93–1.63)	1.32 (1.02–1.71)
Enough education	0.91 (0.85–0.99)	1.03 (0.96–1.10)
Relevance of job	1.05 (0.85–1.31)	0.99 (0.82–1.19)

**Figure 1.**
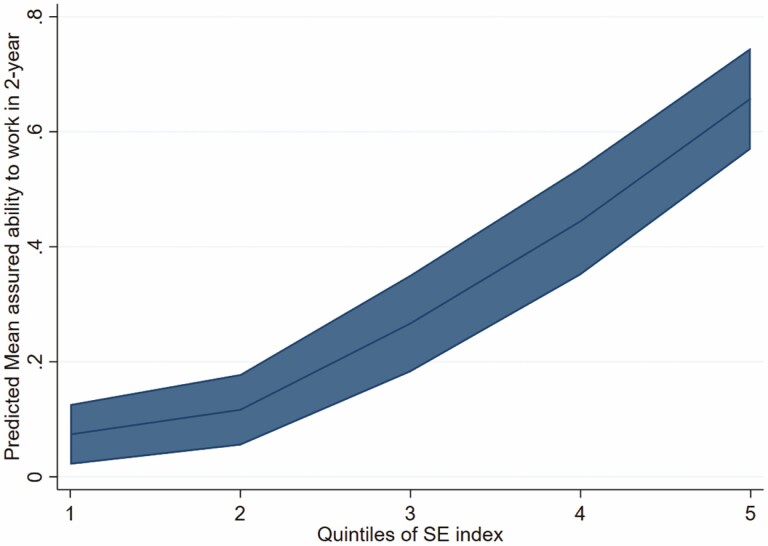
Predicted probability of assured workability in 2 years based on quintiles of SE index in training data set.

Figure 1 shows the predicted probabilities of assured workability for 2 years and their 95% CIs by the quintiles of the SE index. The probability increased linearly with increasing quintiles from 1 to 5.

Table 3 shows expected and observed probability which increases with the quintiles in both the training and validation data sets. The overall observed probability matched the expected probability and numbers very well at all levels of the SE index in both the training and validation data sets. The highest quintiles of the SE index showed the highest observed and expected assured workability in 2 years.

**Table 3.** Expected and observed probability and number of the study population with assured workability in 2 years in training and validation data sets for quintiles of the SE index

Quintiles of SE index				Validation				
	*N*	Observed assured workability in 2 years		*N*	Expected assured workability in 2 years		Observed assured workability in 2 years	
		Probability	Number		Probability	Number	Probability	Number
Q1	95	0.07	7	99	0.16	16	0.16	16
Q2	103	0.12	12	111	0.23	26	0.23	25
Q3	105	0.27	28	111	0.36	40	0.36	40
Q4	108	0.44	48	113	0.53	60	0.53	60
Q5	111	0.66	73	118	0.78	92	0.78	92
Total	522	0.32	168	552	0.42	234	0.42	233

**Figure 2.**
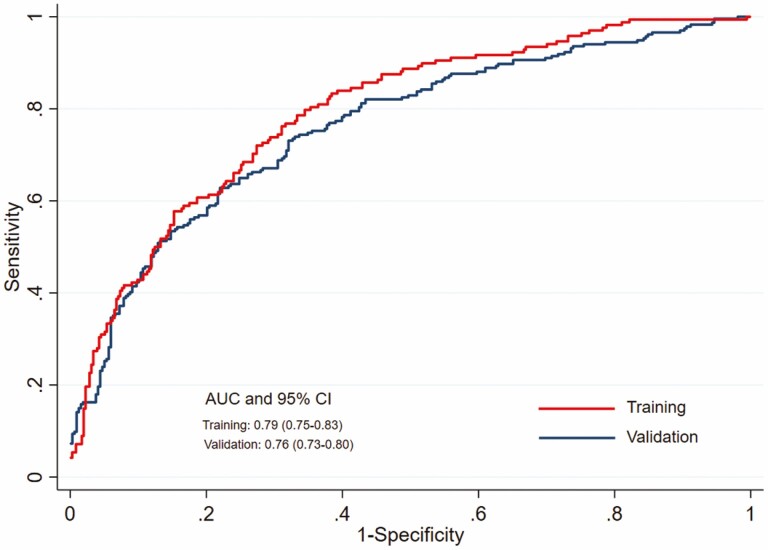
Receiver operating characteristic curves for training and validation data sets.

The predictive ability of the SE index did not substantially differ between the training and validation data sets (Figure 2): AUC for training data: 0.79 (95% CI 0.75–0.83) and AUC for validation data: 0.76 (95% CI 0.73–0.80).

The scoring tool was calculated in the training data set based on the OR of all nine indicators of SE for its practical applicability (Table 4). The score uses a granular scale of 0 to 100, with higher scores indicating higher assured workability with a total score of 100. A scale of 0–100 was used because it is easier to read and interpreted. Among individual indicators, self-rated health and workability and the highest scores of 14 and 13, respectively. Job satisfaction and perceived employment had a score of 12, each. Most of the other indicators had similar importance in the index. The step-by-step process is shown in Table 4. The maximum score was calculated by dividing each odd estimate by the sum of the estimates of all indicators and then multiplied by 100. The weight of the score was calculated by dividing the maximum score by the maximum possible value of the original scale of the measures. The sum of the maximum score of each indicator is 100.

**Table 4.** Score calculation for SE index for predicting assured workability based on log-binomial model among study population in the training data

Indicators		OR	Minimum possible value^a^	Maximum possible value^b^	Maximum score for each indicator^c^	Weight of the score	Calculation method		
	Original scale						Hypothetical value^d^	Multiply by weight of the score	Total score
Self-rated health	0–10	1.41	0	10	14	1.4	8	8 × 1.4	11.2
Work ability	0–10	1.30	0	10	13	1.3	8	8 × 1.3	10.4
Time and resources	0–10	1.06	0	10	10	1	8	8 × 1	8
Recovery after work	0–10	1.07	0	10	10	1	7	7 × 1	7
Job satisfaction	1–5	1.26	1	5	12	2.4	4	4 × 2.4	9.6
Motivation at work	0–10	1.02	0	10	10	1	8	8 × 1	8
Perceived employment	1–4	1.23	1	4	12	3	3	3 × 3	9
Enough education	0–10	0.91	0	10	9	0.9	8	8 × 0.9	7.2
Relevance of job	1–5	1.05	1	5	10	2	4	4 × 2	8
Total			3	74	100				86.4

Weight of the score for each indicator was calculated by dividing the maximum score by the maximum possible value of the original scale of the measures. The score closer to 100 indicates greater employability. The score was calculated by dividing each odd estimate by the sum of the estimates of all indicators and then multiplied by 100. The scores are rounded.

^a^Minimum possible value is the lowest value of the original scale of each indicator from column 2 in the table.

^b^Maximum possible value is the highest value of the original scale of each indicator from column 2 in the table.

^c^Maximum score for each indicator can be obtained by multiplying weight of the score and maximum possible value.

^d^Hypothetical value is the possible response of the employee for each indicator based on the original scale.

## Discussion

We developed an SE index based on nine multidimensional indicators covering individuals’ health, well-being and employability. The SE index correctly predicted the assured workability of the respondents in 2 years, with 16% in the lowest quintile having assured workability in 2 years compared to the 77% in the highest quintile of the SE index. A similar trend was found in the validation data set (10% versus 70%). This suggests that the probability of the workers having assured workability in 2 years increases with increasing levels of the SE index. The predictive ability of the SE index was high and did not differ much between the training and validation data (AUC 0.79 versus 0.76).

Among individual predictors, self-rated health, workability and enough on-the-job training were significantly associated with assured workability in 2 years. Increased odds of having assured workability in 2 years were found in eight of the indicators, except for enough on-the-job training in the training data set. However, the increased odds were significant only for self-rated health and workability, whereas lower odds, but significant association was found for enough on-the-job training. Good self-rated health [20] and opportunities for recovery at work [21] are reported to be associated with workability in previous studies. An earlier Finnish study among timber harvesting professionals also showed the association of recovery from workload with good workability [22]. Although not all associations are statistically significant, higher job satisfaction, motivation to work, perceived employment and high relevance of job were associated with higher odds of having assured workability in 2 years. In line with our findings, a previous study reported that motivation at work was correlated with a greater desire for continued employment upon reaching retirement age [23]. Another study based on the US survey from the Year 2000 reported that persons with a sufficient level of education demonstrate a higher probability of wanting to continue to work in ‘bridge employment’ than persons with a lower education level [24]. Feeling of doing important work, recognition by the employer, performance and appreciation are also reported to be central motivating factors for working at an older age [25].

The score calculated for each indicator based on the regression coefficients was highest for self-rated health and workability, followed by job satisfaction and perceived employment. This means that self-rated health and workability were the most important indicators contributing to the SE index. The score can be used as a practical tool to screen people about their assured workability in future. A previous study shows that sustainable workability during midlife is associated with better survival compared to those with decreasing workability [26]. Therefore workability, alone, is a strong predictor of future health and well-being. Workability, defined as people’s ability to cope with their work demands, is a broad concept and an important form of human capital for workers throughout their careers [27]. It requires continuous processes at workplaces to improve the fit of human resources and the work environment.

We found no previous studies that have developed an index for sustainable employability or employment index to directly compare our findings. However, a few earlier studies have presented similar sets of indicators in their definition of SE which we adapted [3,4].

A large and homogenous sample of older employees was used in this study which is one of its strengths. The study population includes both white- and blue-collar employees, although the majority were in blue-collar tasks. One of the limitations is the use of self-reported single-item measures as the potential indicators of SE. Earlier studies presented sustainable employability models in different populations based on self-reported but multi-item measures [3,4]. Nevertheless, we used valid instruments to measure the indicators of SE which capture satisfactorily three important domains (health, well-being and employability) of employment. We created a dichotomous outcome based on the literature since a dichotomous outcome is common in prediction modelling in clinical research practice [28]. We wanted to predict the good and excellent outcome of 8–10 points, defined as assured future workability in, versus the rest (0–7). Another limitation is that the analysis was done among the sample who had complete information in the outcome. However, some of the indicators of sustainable employability had the response alternatives of ‘don’t know’ or ‘can’t say’ which were removed from the analysis because the SE score calculation does not make sense for these responses.

The duration of the follow-up was short, but we believe that a 2-year follow-up is long enough to follow employees older than 50 years, as the changes in health and co-morbidities are more prominent at older ages [29,30]. Moreover, the outcome ‘assured workability in 2 years’ measures the perspective of the next 2 years at the follow-up and that makes a total of 4 years of follow-up, in principle. We internally validated our SE index model in a different set of the same population. External validation of this tool by replicating the model in a sample of older employees from a different population is required before it can be used in practice. We studied a representative sample of older Finnish postal service employees. There was almost no difference between the respondents and those excluded from the analysis in terms of gender (60% versus 57% male) and age (mean 56.43 (SD: 3.39) versus 56.34 (SD: 3.58)). Therefore, the findings may be generalizable beyond the population of the current study. The study population includes both white- and blue-collar employees; however, the majority worked in blue-collar tasks. We only had a 46% response rate at the baseline which means that there may be a selection or response bias. Nevertheless, a response rate of 46% is considered good, and even lower response rates need not necessarily lead to biased results [31].

In conclusion, we developed and internally validated the SE index among a sample of older employees. The SE index was able to distinguish between employees with and without assured workability in 2 years. We developed a scoring method, which can be used to calculate the potentiality of future employability among late midlife employees, meaning that those with high SE scores have assured employability. Employers might screen their employees and it may ultimately help them to conduct feasible programmes targeting employees with suboptimal employability to improve employability. Following external validation, the tool may be used by occupational healthcare, epidemiological research and government programmes as a screening tool for employability among older employees.
